# Hybrid Soft Ballistic Panel Packages with Integrated Graphene-Modified Para-Aramid Fabric Layers in Combinations with the Different Ballistic Kevlar Textiles

**DOI:** 10.3390/polym16152106

**Published:** 2024-07-24

**Authors:** Silvija Kukle, Aleksandrs Valisevskis, Ugis Briedis, Ilze Balgale, Ieva Bake

**Affiliations:** Institute of Architecture and Design, Riga Technical University, 1048 Riga, Latvia; silvija.kukle@rtu.lv (S.K.); aleksandrs.valisevskis@rtu.lv (A.V.); ilze.balgale@rtu.lv (I.B.); ieva.bake@rtu.lv (I.B.)

**Keywords:** Kevlar fibre, graphene-modified, para-aramid fabrics, ballistic protection, pressure sensor

## Abstract

The purpose of the research discussed in this article is to explore the possibility of creating hybrid soft ballistic panel (BP) package variants by integrating into their composition layers of graphene-modified para-aramid fabrics in combinations with the different ballistic Kevlar textiles to improve the durability of the first layers of the soft ballistic panel. To address this goal, the liquid-phase exfoliation (LPE) method was used for integrating dispersions into composites to solve a number of topical problems in the stages of the technological sequence development of processing methods and optimizing processing parameters in accordance with the processing specifics of aramid textiles to achieve the desired properties of modified ballistic fabric, including the provision of coating adhesion to the surface to be modified. To test the results, ballistic experiments were performed and the back-face signature (BFS) of bullet impact on a backing material was analysed according to standards. Bullet impacts on the first ballistic protective fabric layers were also studied.

## 1. Introduction

Modern military operations, technology-driven warfare strategies and everyday used weapons and ammunition demand the development of sophisticated ballistic protection armour systems that are highly energy-absorbing, flexible, lightweight and resistant to damage [1]. Ballistic body armours made from high-performance fibres, such as para-aramid (Kevlar^®^, Twaron^®^), and ultra-high molecular weight polyethylene (UHMWPE) fibres, are widely used in personnel ballistic protective armours for military and law enforcement applications, due to their flexibility and light weight [2]. Traditionally, a soft ballistic panel (BP) is manufactured by layering numerous fabric layers with their total weight reaching 3 to 5 kg [3]. The propagation speed of the shock wave created on the ballistic plane following ballistic impact is proportional to the energy absorption capabilities of fabric layers and is significant from a ballistic standpoint. 

However, according to previous studies, each fabric layer at different positions of a multilayer panel plays different roles in ballistic resistance, considering that the first layer absorbs the most energy and the absorbed energy amount gradually decreases in the successive layers [4,5,6]. Long-term targeted studies have shown that when a multilayer panel is under ballistic impact [7], energy absorption of each layer is increased from the front layer to the peak value at the last perforated layer and then gradually decreases in the following back layers of the BP. This pattern is not influenced by the total number of layers in the BP. When increasing the threat level, only the position of the peak value of energy absorption with the last perforated layer is shifted towards the back of the BP [6,8,9,10]. 

Due to different roles of each layer in ballistic resistance, layering up the same fabrics in a panel cannot be the most efficient method for ballistic performance. For the perforated ballistic panels, it has been found that the front layers have smaller transverse deformation but with higher stress concentration at the vicinity of the impact point. The rear layers, on the other hand, exhibit larger transverse deformation and wider stress distribution. More fabric materials in the rear layers are engaged in the strain zone than that in the front layers, indicating different ways of energy absorption between the front and rear layer groups [8,9]. The studies carried out have aroused interest in hybrid ballistic body armour design, and many patents and commercial hybrid products have been efficient in providing superior ballistic performances and reductions in weight [11,12,13]. Different materials are mixed in a panel system to best utilize their qualities. Many studies have shown that hybridization is an efficient method for improving ballistic performance and reducing the weight of ballistic protection items [14,15,16]. Performance is considerably influenced by the materials’ qualities of layers and the sequence of layering up. Finite element (FE) modelling and ballistics tests have shown that when fabrics are layered up in a panel, a multilayer panel can be purposefully divided into three groups. 

In the first group, when a projectile impacts on a BP, the stress waves generated on the impact area propagate down the axis of the primary yarns and increase sharply during less than 10 µs. Due to the very rapid failure of the layers’ structure, the stress wave cannot propagate widely. In addition, the transverse deformation area of layers in this group localise mainly around the edge of the projectile. The transverse transmission of impact energy from primary yarns is less noticeable. Such ballistic characteristics of the first group indicate that some tough materials should be combined in the BP striking face in order to sustain them longer before fracture under impact stress [10]. According to the FE simulation and ballistic test results, the authors concluded that if the proper materials were placed in the front sensitive region, a synergistic hybrid effect may have manifested in the improvement of energy absorption of the hybrid panel for a given areal density of ballistic packages [17].

The second group contains fabric layers close to the last perforated layer. FE results show that the fabric layer has a longer interaction time (around 20 µs) with the projectile before fabric fracture than that of front layers. The stress wave can propagate over a wider area from the impact point to the edge of fabric before fabric fracture. Lightweight fabrics obtained by using fine yarns or reducing the weave density can be combined in this group to apply higher energy absorption capacity [7].

In the third group, back layers cannot be perforated and only produce transverse deformation until the projectile stops. Due to the attenuated impact force, the stress magnitude becomes lower, and the transverse deflection is gradually decreased. This means that material properties of the back layers cannot be fully used during the ballistic impact process, and the energy absorption efficiency of the fabric layers in this group is low. At the same time, according to ballistics tests, these layers in the back group play important roles in minimizing the back-face signature (BFS) of the panel. Therefore, materials that possess high stress wave velocity resulting in lower BFS should be combined in this group [10].

Different parameters affect ballistic resistance, such as fibre and yarn tensile modulus and other properties [6], fabric construction, fabric area weight and fabric ply number used in protective structures. Apart from these parameters, bullet speed, shooting angle, bullet geometry and boundary conditions are other parameters affecting impact behaviour [18]. 

Another parameter affecting ballistic properties is the friction between yarns, which plays an important role in the response of aramid fabrics to impact, both in a direct and indirect way. The direct effect is reflected in an increase in the energy dissipation of the fabric when the yarns begin to move, either through sliding, stretching or reorienting the yarn. The indirect effect is reflected in the way the loads are transferred and redistributed between neighbouring yarns [12,19]. Other studies have numerically analysed and compared the influence of the friction between fabric and projectile, and between the yarns during an impact [20], concluding that the latter interaction plays the most important role in the response of the fabric to impact. This point was investigated by several researchers [13,19,20]. Bai et al. [19] showed that a moderate change in friction force between the yarns caused a significant change in ballistic performance and bringing the friction force between the yarns to the highest level caused more energy-propagating ability in the fabrics. Cunnif [21] found that ballistic performance decreased in loose fabric structures (i.e., lower weft and warp densities) as well as with lower friction force between the yarns. Duan et al. [22] pointed out that the frictional forces help to protect fabric structure during ballistic impact. Other studies found if the inter-yarn friction is greater, the velocities of the transverse stress waves in the woven fabrics are higher, and the distributions of impact energy from the primary yarns of the fabrics to the secondary yarns of the fabrics are more effective. In previous studies [23,24], yarn surfaces were treated with silica colloidal to increase frictional forces between the yarns; the ballistic performance of fabrics increased by using these yarns. 

Theoretical analysis by means of FE simulation show that inter-yarn friction would significantly affect the ballistic behaviours of yarns in fabrics. More inter-yarn friction leads to more impact energy shared by secondary yarns, thus alleviating the loads in primary yarns and prolonging the failure of primary yarns [25]. In addition, at higher levels of inter-yarn friction, the structure of the fabric can be kept much more stable, less slippage of primary yarns occurs, and there is more resistance force to the projectile. However, much higher inter-yarn friction, somewhat beyond a coefficient of static friction (CSF) of 0.8 and a coefficient of kinetic friction (CKF) of 0.75, is counterproductive because it will cause the stress to be concentrated more on primary yarns, resulting in earlier failure of the fabric. Combined effects caused by inter-yarn friction make the failure time of the fabric fluctuate with the variation in inter-yarn friction. Impregnation of fabric with shear thickening fluid (STF) is the mostly used method to increase the frictional force of a single yarn in the fabric, which consequently increases the apparent modulus of the yarn. It has also been established that when the STF-impregnated fabrics were laminated behind the neat Kevlar layers, the BFS decreased compared to the panel of all neat fabrics [13]. Chu et al. [25,26] used sol–gel treatment of aramid yarn to increase inter-yarn friction. In study [27], graphene nano-platelets (GNPs) embedded in a high-density polyethylene (HDPE) film was used to form a multi-layer laminated panel. Mechanical, dynamic–mechanical and ballistic impacts of HDPE-0.5% GNP showed the highest energy dissipation. Several laminated plates of graphene-based nanocomposites, composed of a polyester resin matrix doped with few layers of pristine graphene and reinforced with a fibreglass woven fabric, using doping percentages ranging between 0.25% and 1% in weight, were produced [28]. Test results support the viability of the development of new graphene-based nanocomposites with improved mechanical [29] and ballistic protection properties for security and defence applications. 

Despite a comprehensive interest from researchers focused on identifying opportunities to improve the performance of materials/surfaces/objects at the macro level with the excellent physical and mechanical properties of graphene, the studies conducted to date have addressed individual problems, related mainly to the development of graphene extraction “top-down” and “bottom-up” methods and corresponding laboratory technologies, and have explored the possibilities of moving towards the assignment of predictable graphene sheet/layer properties and solutions for the creation of stable graphene dispersions [30]. Our understanding is gradually growing, and theoretical foundations for process descriptions are being formed. The small number of studies carried out so far on the integration of graphene into BP have been complemented by previous studies [28,29].

Kinetic energy of the projectile impacting the protective target must be absorbed by the target through different kinds of damage- and energy-absorbing mechanisms [13,31]. It has been assumed that by increasing the resistance ability of BP to the impact kinetic energy of the projectile with a functional nanostructured graphene coating used to improve inter-yarn friction of the fabric layers of the ballistic package, the overall performance of the ballistic panel increases, and the number of fabric layers can be reduced, thereby reducing the weight and the BFS. Within this framework, several technological sequences have been developed to obtain graphene dispersions by liquid phase exfoliation (LPE) of pristine graphite flakes and following graphene deposition on Kevlar woven ballistic fabric [28,29].

Fabric ply number used in ballistic panels is the most important parameter affecting the trauma depth and diameter. An increase in fabric ply number caused a decrease in trauma depth and diameter [13]. The purpose of the research discussed in this article is to explore the possibility of creating hybrid soft BP package variants by integrating into their composition layers of graphene-modified para-aramid fabrics in combinations with different ballistic Kevlar textiles and/or composites.

## 2. Materials and Methods

### 2.1. Theoretical Methods

#### Technological Sequence to Obtain Graphene-Modified Kevlar Fabric

**Figure 1:**
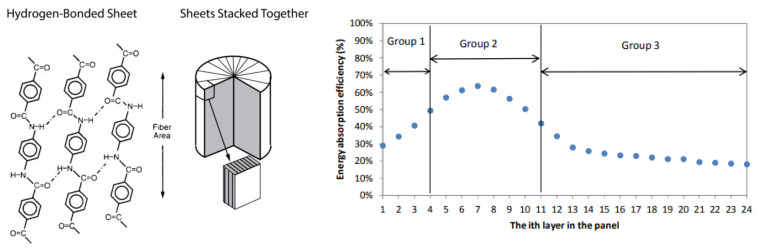
Rod-Like Kevlar fibre structure, showing the radial stacking of hydrogen-bonded sheets (**left**) (Technical Guide for Kevlar^®^ Aramid Fiber). Energy absorption efficiency R of Twaron woven ballistic panel (BP) (**right**) [7].

The polymer chains of para-aramid are linked into a locally planar structure by hydrogen bonds across the chains, with transversal strength considerably weaker than longitudinal strength (Figure 1, left).

In paper published more than 10 years ago [32], the authors proposed a hypothesis that introducing an outer enveloping layer of graphene, linked to polymer chains by strong chemical bonds, may significantly strengthen Kevlar fibre with respect to transversal deformations. To support the hypothesis, a 2D linear elasticity model to predict the mechanical properties of Kevlar fibre covered by a graphene outer shell attached to the Kevlar fibre with a chemical bond was developed. The authors proposed that the outer layer of graphene can be formed by soaking the Kevlar fibre in the graphene dispersion of the N-methylpyrrolidone (NMP) solvent and creating a composite structure from it in such a way that the graphene layer covers the Kevlar fibre. It was hypothesized that graphene could be incorporated into swollen Kevlar fibres by providing chemical functionalization of graphene and Kevlar by modifying fibrous surface functional (e.g., carboxyl) groups, and the obtained outer graphene layer covering the Kevlar fibre could stabilize radially stacked hydrogen-bonded flat sheets, improving the Kevlar fibre resistance against transverse deformations. In the resulting 2D linear elasticity model, taking into account the real ratios of Jung’s module to the Poisson coefficient for transverse deformations, the reinforcing effect may begin to occur when the radius of the outer solid shell is about 4 % of the radius of the Kevlar fibre. As the radius of commercially available Kevlar fibre is about 5–6 µm, the model predicted that approximately 240 nm of functional multilayer graphene would be required to double the yield strength [32]. Although the established 2D linear elasticity model predicted a promising solution, its implementation has thus far been delayed due to its complexity.

**Figure 2:**
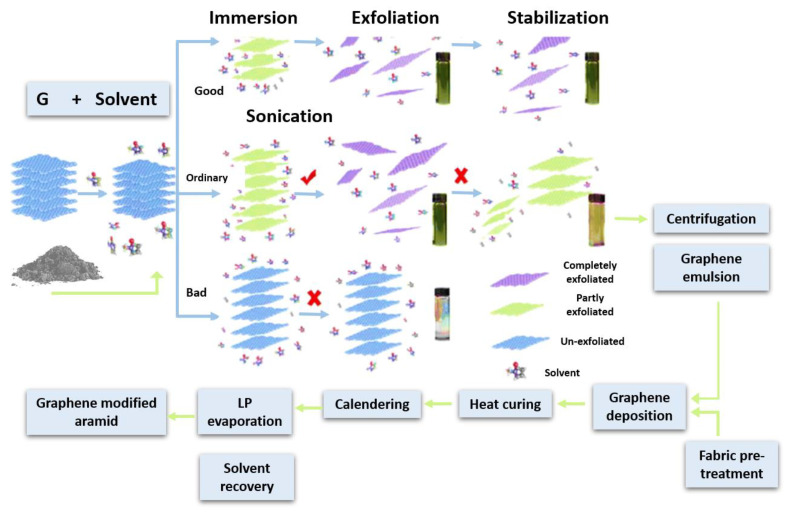
Technological sequence to obtain graphene-modified Kevlar fabric, 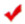
—separated for dispersion, 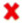
—separated in sediments.

A technological sequence to obtain graphene-modified para-aramid fabric following the predictions discussed above and taking into account the results of modelling has been developed and is shown in Figure 2. The graphene-modified para-aramid fabrics obtained by using this technological sequence have been applied as hybrid BP package layers of the first group (Figure 1, right) due to the reinforcing effect of the graphene coating.

LPE is included in the technological sequence of “graphite-graphene dispersion—textile coating—properties of modified para-aramid ballistic fabric—integration of the modified fabric layers into the BP” (Figure 2) to solve a number of topical problems in the stages of the technological sequence development of processing methods. It is also used to optimize processing parameters in accordance with the processing specifics of aramid textiles to achieve the desired properties of modified ballistic fabrics, including the provision of coating adhesion to the surface to be modified.

Liquid phase exfoliation (LPE) involves three main steps: the dispersion of graphite in a liquid medium with solvent; the exfoliation of dispersion via sonication; and dispersion stabilization (Figure 2) [33]. Effective solvents for graphite exfoliation have non-zero polarity δP and hydrogen bonding δH values despite the non-polar nature of graphene, and all three Hansen solubility parameters are essential when describing the affinity between solvent and solute. Cyrene presents a close match to the parameters δD, δH and δP of graphene [34]. Despite relatively successful initial results [28,29,35], the authors failed to obtain a stable Cyrene-based emulsion for modifying Kevlar with graphene and ensuring adhesion to the fibrous surface of Kevlar fibres.

Table 1 shows that solvent candidate N,N-Dimethylacetamide (DMAc) meets the requirements of all three parallel performance criteria [33]: the solvent surface tension falls within the designated range of 38.2 ± 6, the Hansen distance between target and solvent is lower than 6.5 MPa^0.5^, and the density/viscosity ratio (1.04) is low. Another problem when thinking about integrating dispersions into composites has to do with the fact that a polar aprotic solvent is incapable of performing hydrogen bonding. To solve this problem, two other constituents were added to form the liquid phase: triethanolamine (TEA) (Table 1) and trisodium citrate. TEA presents close δD and δP matches to the graphene and, as a nucleophile, shows much higher δH, which suggests the ability to more easily bond to the desired molecule in the coating process. As an amine, TEA is able to accept hydrogen to form hydroxide and conjugate acid. This raises the pH of the solution, and, as a surfactant, TEA can reduce the interphase tension in the mixture or solution, preventing the emulsion from layering or the deposition of compounds from the solution [36].

Creating the liquid medium for the graphene LPE from graphite, Du et al. used solvents. NMP, N,N-dimethylformamide (DMF) and dimethyl sulphoxide (DMSO) were supplemented with the organic salts, namely potassium sodium tartrate (KNaC_4_H_4_O_6_·4H_2_O), sodium tartrate (Na_2_C_4_H_4_O_6_·2H_2_O), sodium citrate (Na_3_C_6_H_5_O_7_·2H_2_O), edetate disodium (Na_2_C_10_H_14_N_2_O_8_·2H_2_O) and sodium hydroxide (NaOH); as a result, they found that DMSO in the presence of sodium citrate was the best medium [37]. We present an efficient LPE route based on organic salt trisodium citrate (Table 2) [38] assisted exfoliation of pristine graphite in organic solvent DMAc-based liquid media, as organic salts can markedly improve exfoliation efficiencies and allow increasing the graphene concentration in dispersion. The choice of NaCi for inclusion in the formation of liquid media also takes into account, among other things, properties of NaCi such as the ability to form a porous matrix, and to be a hydrogen bond acceptor (7, white dots in the structure scheme) and donor (1) (Table 2). This is important when, in next steps of the technological sequence (Figure 2), it has to form strong adhesion to the surface of aramid fibres with the formation of chemical bonds. 

### 2.2. Materials and Methods 

Military ballistic fabrics used included KevlarKM2 600D and KevlarKM2+ 440 type 310L (SAATI), Uni-Directional aramid UD ballistic fabric (Skarr Armor fabric (Skarr Armor, 412 N Main St Suite 100, Buffalo, WY, USA)) composed of two layers of fibres, area density 215 ± 10 g/m^2^; HPPE Bulletproof Fabric/SB130 UHMWPE UD made from 0/90/0/90 (4) Uni-directional structure high-tenacity UHMWPE fibres, tenacity 36cN/dtex for body armour manufacturing (Model Number SB130), area density130 g/m^2^ (supplied by Skarr Armor). Du Pont™ Kevlar^®^ XP™ K520 consists of two layers of fibres in +45°/−45° orientations that provide the required stopping power to address standard ballistic threats to body armour with fewer layers (fabric for tests was received from DuPont European Technical Centre in Meyrin, Switzerland). Airloy^®^ HR was supplied by Aerogel Technologies, LLC, (270 Dorchester Ave, Boston, MA, USA).

Experimental dispersions were obtained by mixing graphite flakes (>99%, carbon basis, 325-mesh particle size, natural, Sigma-Aldrich (3050 Spruce St Saint Louis, MO, USA) with the solvent Cyrene (Dihydrolevoglucosenone Cyrene™, Sigma-Aldrich) in a concentration of 25 mg/mL or DMAc 100 mg/mL, which were further processed according to previous methods [28,29], creating five variants that differ depending on when TEA (triethanolamine reagent grade 98%, Sigma-Aldrich) was added to the dispersion (Table 2 and Table 3). 

The proposed method utilizes one-step (60 min) or two-step (60 and 30 min, respective) sonication (Hielscher Ultrasonic (Oderstraße 53, Teltow, Germany) Processor UP200H) and re-dispersion of sediment, replacing high-speed centrifugation with medium speed and reducing the speed by about (31–50)% in the second round and during sediment dispersion processing (Centrifuge Ohaus (Heuwinkelstrasse 38606 Nänikon Switzerland) Frontier FC5816; speed range: 200–8000 rpm). Regarding the chosen processing modes, it should be noted that if higher centrifuge revolutions are applied, the lateral dimensions of flakes decrease, while in the case of a lower rotational speed, the thickness of the exfoliated graphene layers increases. Sonication parameters used were: cycle 0.5 and amplitude 80%, with the sonotrodes S7 or S14, depending on the volume to be processed.

Viscosity measurements were carried out with a viscometer (Lovis 2000 M/ME; Anton Paar (Anton-Paar-Straße 20 Graz, Steiermark, Austria) at 20 °C. Graphene particle size analysis and ζ-potential measurements were conducted with a LitesizerTM 500 instrument (Anton Paar) using appropriate software. Particle size measurements were performed on undiluted samples, while ζ-potential measurements were made with samples at a 1:30 dilutions. Samples for the tests were taken with a disposable pipette from the upper part of the tube before allowing the samples to settle for at least one hour. Glass cuvettes were used for measurements. The dispersion cells were in a thermostatic chamber at 20 °C during the test process. A series of measurements were carried out for each sample, from which the distribution of the average dimensions and ζ-potential were calculated. A scanning electron microscope (SEM) Helios 5 UX (In-Vision Technologies AG, Industriestrasse 9, Guntramsdorf, Vienna, Austria) was used to investigate modified surface morphology and measure particle lateral sizes and size distributions. Measurements were taken at the Institute of Solid State Physics, University of Latvia. For the sizing of graphene flakes from SEM micrographs, three series of lateral size measurements were performed for each variant, with 100 measures per series. The results of the measurements using ImageJ software V1.8.0 are shown in graphs of the lateral size distributions of the flakes. Values of the parameters characterizing the distribution of the variants are also compared.

The colours of pristine and modified Kevlar fabric samples were visualized and quantified by using the CIELAB colour space. The colour points in the colour space L*, a*, b* were determined by an X-Rite Pantone Capsure RM200 spectrocolorimeter (X-Rite Co., Ltd., 4300 44th St. SE Grand Rapids, MI, USA) as averages of 10 measurements. For colour analysis, we used online converters: CIELab to Pantone: NIX. Colour Sensor—Free Colour Converter; CIELab to Pantone and Pantone ID calculator: Calculatormix (this calculator converts LAB colour codes into the closest Pantone value); Pantone to CIELab: icolorpalette Colour Info Converter.

### 2.3. Ballistic Experiment Measurements

In the ballistic experiments, textile pressure sensors of the matrix type and knitted sensors developed by the authors were used to determine the point of impact of the bullet. The use of this sensor makes it possible to analyse the impact of the bullet on the soft package fabric layers at the moment of impact—especially in the first 20 microseconds. The sensor data show the change in electrical resistance (kΩ) at the moment of bullet impact.

**Figure 3:**
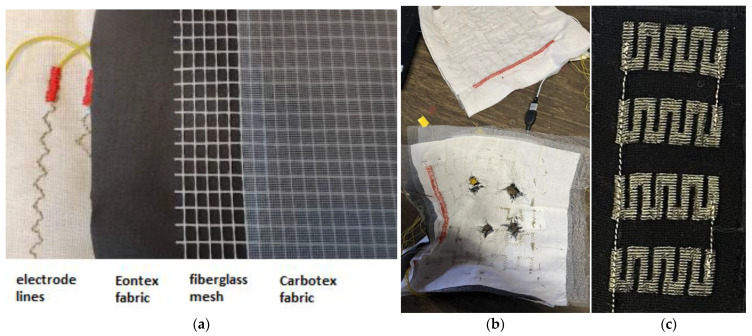
(**a**) Layers of matrix sensor, (**b**) matrix sensors before shooting (**top**) and after shooting (**bottom**), and (**c**) knitted sensor.

The dimensions of the sensors are 225 × 225 mm. The matrix sensor consists of 5 × 5 electrodes embroidered with electroconductive thread on layers of outer cotton fabric (horizontally and vertically) (Figure 3a,b). The middle layer consists of a combination of electrically conductive fabrics (EeonTex LTT-SLPA 60 kOhm (made by Eeonyx Corp. (750 Belmont Way, Pinole, CA, USA)) and Sefar Carbotex (Sefar AG, Hinterbissaustrasse 12, Heiden, Switzerland)) with fibreglass mesh. The selection of electro-conductive fabrics for the pressure sensor was based on previous studies conducted on piezoresistive materials [39,40]. Two structurally and functionally distinct types of materials were selected (knitted coated fabric and woven fabric with composite threads). All of the materials are textile-based, so they can be easily tailored and integrated into textile structures, such as vests or jackets. EeonTex LTT-SLPA 60 kOhm is a bi-directionally stretchable elastic knitted fabric containing 72% nylon and 28% spandex, and is coated with a proprietary conductive formulation, based on conductive polymers. Its area density is 163 g/m^2^ and it has a thickness of 0.38 mm. Sefar Carbotex is woven fabric, composed of polyester threads in warp direction and unidirectional weft threads, made of carbon-loaded composite material, which alternate with non-conductive polyester threads. In previous studies, where several commercially available piezoresistive fabrics were analysed, the authors concluded that multiple layers of EeonTex and combinations of this material with Carbotex fabrics with an intermediate fibreglass mesh layer proved to be better suited for determining bullet energy [40].

The sensor was made with embroidery technology with triple zigzag stitches 3 mm in length and 3 mm in width (3 × 3 stitch pattern); a 1 mm stitch length was chosen for the embroidered layer. Madeira HC-12 (made by Madeira, Rudolf Schmidt KG Zinkmattenstrasse 38, Freiburg, Germany) electro-conductive thread inserted into a no. 90 needle was used to make conductive traces. Polyester thread (no. 40) was used in the bobbin. A lightweight cotton fabric (100 g/m^2^ density) was used as the base material. The terminal end of the sensor was connected to a copper wire (0.5 mm in diameter), using an embroidery machine (zigzag stitch, width 3 mm, density 4.5 lines/mm, fixing length 10 mm). The sensor pattern was programmed in the embroidery CAM software Brother PE Design V.8 (made by Brother Industries, Ltd, 15-1, Naeshiro-cho, Mizuho-ku, Nagoya, Japan) and embroidered with a computerized embroidery machine (Brother PR-600II made by Brother Industries, Ltd). Embroidery was completed by placing the cloth in a 100 × 100 mm embroidery frame at a speed of 600 rpm.

An electronic system based on a SAM D21 32-bit microcontroller (Microchip Technology Inc. 2355 West Chandler Blvd. Chandler, Arizona, USA) was built for signal recording and pre-processing. The system measured resistance with a sampling rate of around 80 kHz and has a built-in threshold for impact detection. Upon detection of the impact, the data points right before and after the impact were transferred to a computer via a serial communication port. Threshold activation ensured automatic data alignment and simplified further data analysis. During the ballistic experiments, the pressure sensors were placed behind the ballistic panel.

**Figure 4:**
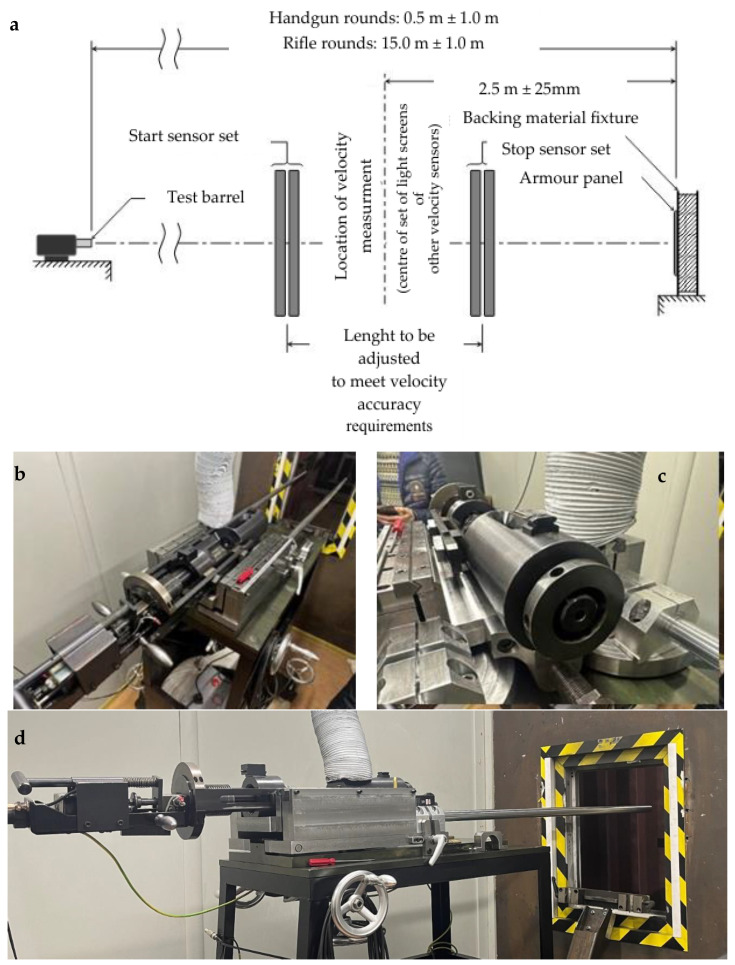
Test range configuration (**a**); test stand with a test barrel (**b**–**d**).

Ballistic tests were performed in the ballistic testing laboratory of SIA Vairog EU (Pils Str 9 - 5, Riga Latvia). The test range configuration is shown in the picture Figure 4a. Test ammunition was (9 × 19) mm with 8 g (124 gr) full metal jacket bullet.

The laboratory equipment used was as follows: test stand (Figure 4b–d) with a test barrel—Twist Rate: 1 in 254 mm, barrel length: 199 mm, Start Sensor Test Equipment, Stop Sensor Test Equipment, Handgun Rounds: 5.0 m ± 0.5 m. Experiment conditions: indoor temperature: 23 °C, outside temperature: 3 °C, air pressure: 1028 hPa.

To obtain and analyse the back-face signature (BFS) of bullet impact, a standard backing material was used: oil- and wax-based modelling clay, which is extra smooth and flowable, does not contain water, never hardens, is not adversely affected by heat and contains sulphur. The ballistic experiment used NIJ Standard 0101.07 Ballistic Resistance of Body Armor [41] with IIIA level of ballistic performance and was conducted in a certified indoor laboratory.

After shooting, the backing material was struck to return the surface to a flat configuration. The armour panel was manipulated by hand so that any deformations in the armour were smoothed out.

During the test with the laboratory equipment, we recorded:

1. Bullet speed (m/s) range: 425.73–440.31 m/s, which is in accordance with the standard (velocity of 430 ± 9.1 m/s).
2. Initial energy of the bullet (J)—in the range 728.18–778.91 J.
3. After the shooting, the back-face signature (BFS) was measured by callipers, and the measurements depending on the ballistic panel used were in the range: 17.03–39.02 mm, which is in accordance with the standard NIJ 0101.07 (max value is 44 mm).

According to the standard, the measured backface signatures from a P-BFS test for new armour were analysed to determine if the armour will provide adequate protection against blunt trauma behind the armour. The requirements specify that either all measured BFS depths due to fair hits shall be 44 mm or less, or if any BFS depth exceeds 44 mm, there shall be 95% confidence that 80% of all BFS depths will be 44 mm or less. Since at this stage of research, the ballistic Kevlar fabrics were modified with graphene coating, fragmentation resistance tests were not performed.

## 3. Results and Discussion

### 3.1. Conformity Assessment of Emulsion

Comparative analysis of hydrodynamic diameters of the G particles measured together with the particle enveloping layer in solution (Table 4) shows that diameter values of both dimethylacetamide (DAMc)-based emulsion vary in a narrow interval, with a relative error of 2% DTC and 3% DTC-b, respectively. Mean particle diameters of dispersion of recovered sediments DCT-b exceeded two times the corresponding DMAc value, which is explained both by higher emulsion viscosity and by the deliberately reduced number of centrifuge spins with the aim of increasing the thickness of the fibre coating with graphene. In principle, an increase in viscosity would be beneficial for the LPE process, increasing the exfoliation yield and decreasing the defect and sedimentation rate. However, an excessive viscosity favours the stable suspension of large particles and agglomerates during the centrifugation step, thus preventing their separation from thinner and lighter flakes. It is difficult to regulate viscosity in the liquid medium of the three components, given that the change in the relative percentage of components has a significant impact on the functional properties of the emulsion. It is much easier to adjust the desired size distribution of graphene flakes by adjusting the sonication time and/or the rotation speed of the centrifuge or processing time. The emulsions from residues DTC-b and Cyrene based emulsion CTC-b considered in Table 4 had a suitable spin rotational speed of 1200 min^−1^ compared to the centrifuge rotational speed of 3200 min^−1^ applied in the DTC and CTC variant processing; this difference is considered to be the main reason for such significant differences in average diameters of DTC-b, which were less pronounced in CTC-b but still had extremely wide confidence intervals of the mean diameter. The values of the DTC and DTC-b emulsions fall within the range of 2–3 %. Relative errors of 37% (CTC-b) and 57% (polydispersity index) are many times higher than the corresponding DTC and DTC-b indicators. While polydispersity indices of CTC emulsion and CTC-b have a corresponding high score of more than 2x, both emulsions are rated as being of little relevance for future use. 

The polydispersity index (PDI), which characterizes the particle size distribution width, of DTC and DTC-d were 18% and 22% (Table 4), respectively, exceed 10%, at which dispersions according to standards [42] are considered monodispersed, but are far from 70%, at which they are considered highly polydispersed. Given that the PDI values of both emulsions also suggest that the emulsions of the graphite-flake diameters are influenced relatively little by the variation in spin revolutions of the centrifuge within broad limits, it can be considered that with the choice of centrifuge rotation speed, hydrodynamic diameter distribution parameters can be regulated. 

The zeta (ξ) potential, which characterizes the electrochemical balance at the particle–liquid interface by measuring the magnitude of electrostatic repulsion/attraction between particles, has become one of the basic parameters for assessing the stability of colloidal particles. DTC and DTC-b ξ-potential values indicate that both emulsions are considered stable, which has also been confirmed in practical applications with the long emulsion shelf-life.

Although the average diameter values of Cyrene-based emulsions are comparable to those discussed above, the confidence intervals of the diameters, and thus the confidence intervals of the PDI, are large (Table 4). Since polydispersity may occur due to the particle size distribution in the sample, as well as agglomeration or aggregation of the sample during the isolation or analysis of the sample, it can be concluded that emulsions contain a lot of partly exfoliated, large-size G flakes. During the measurement process of ξ-potential, it emerged that the both Cyrene-based emulsions did not develop ξ-potential, which explains the instability of these dispersions found in practice. As the stability of both Cyrene-base dispersions was assessed as very low for aramid modification, DMAc-based emulsions were used for aramid fabric modification.

### 3.2. Development of Graphene-Modified Para-Aramid Fabrics

**Figure 5:**
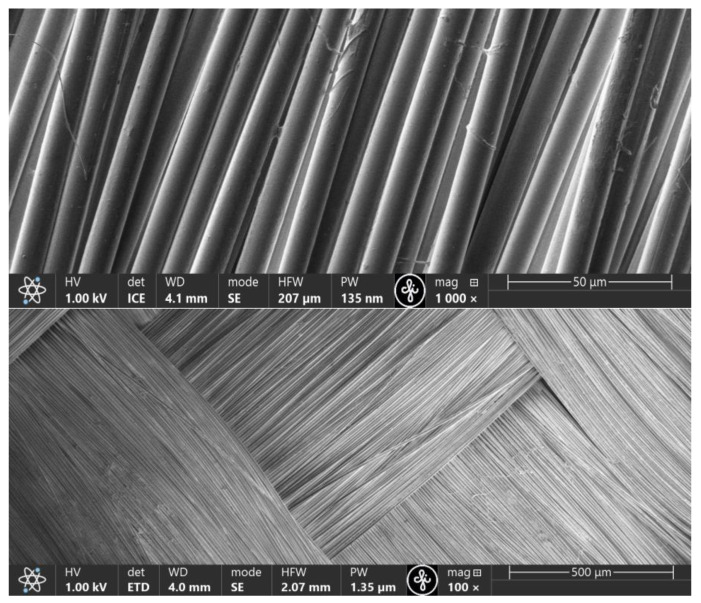
SEM micrographs of unmodified KM2 600D fabric.

**Figure 6:**
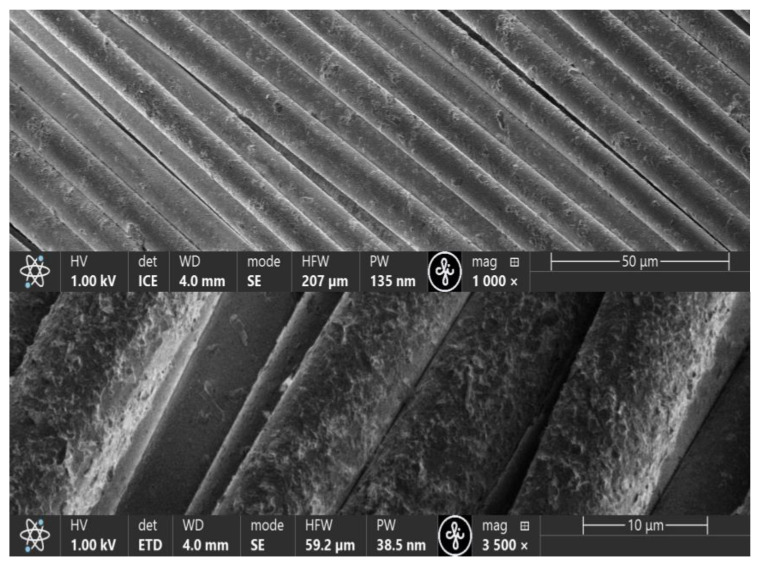
SEM micrographs of modified fabric fibre surfaces to which three coating layers were applied.

Emulsions containing exfoliated graphene flakes were applied to the purified KevlarKM2 600D or KevlarKM2+ 440 type 310L fabric samples according to the technological sequence (Figure 2). Given that the surfaces of the fibres forming the fabric was very smooth (Figure 5) after applying the emulsion, the samples were subjected to 1 h of prolonged aging at a temperature of 60 °C to form chemical bonds with the fibrous surface. To ensure the desired properties of fibre surface coatings, the deposition process was carried out repeatedly. The micrographs in Figure 6 show that in the process of fabric modification, graphene flakes form a scaly coating on the surface of the fibres, making the surface rough, thereby increasing the inter-yarn friction. 

**Figure 7:**
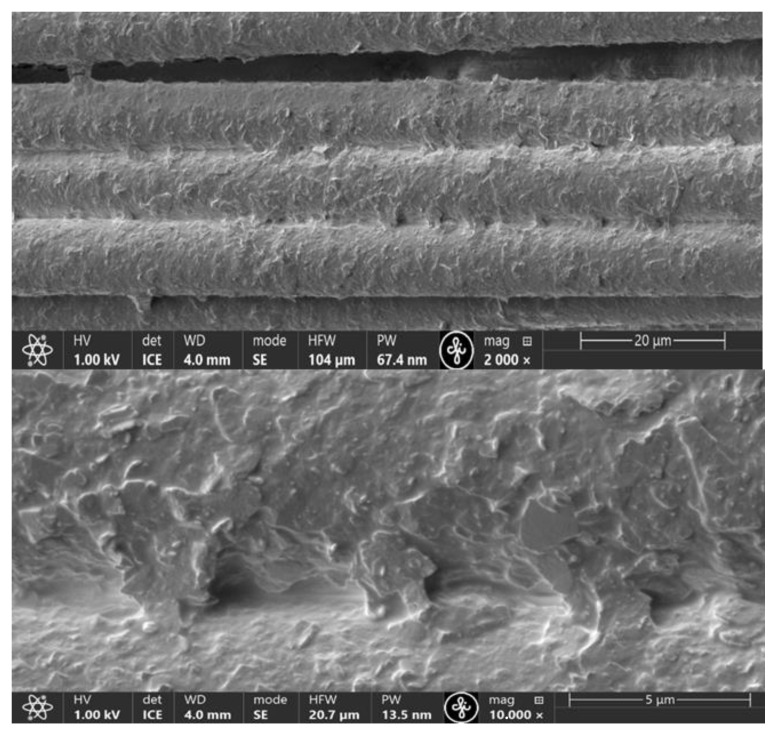
SEM micrographs of modified fabric fibre surfaces to which five coating layers were applied.

By increasing the number of coating layers between closely adjacent fibres, graphene flake bridges are formed, and the coating becomes continuous (Figure 7). 

**Figure 8:**
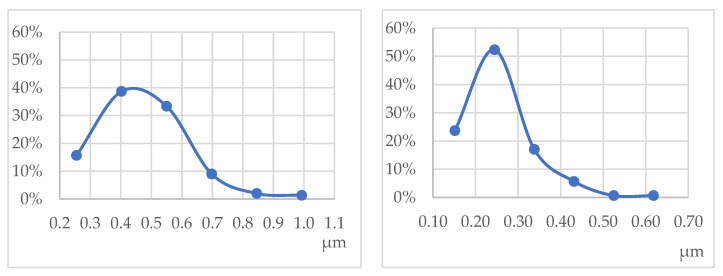
Lateral size distribution of graphene flakes obtained from micrographs measured at magnifications of 2000× (**left**) and 10,000× (**right**).

Comparing graphene lateral size distribution graphs, it is seen that the lateral distribution of deposited graphene flakes depends on magnification, as only flakes that can be seen are measurable from micrographs (Figure 8). Compared to hydrodynamic diameter measurements in an emulsion (Table 4), the most appropriate is the distribution obtained by measuring lateral dimensions from micrographs at 10,000-fold magnification. For 300 measurements from the micrograph of the modified sample, the estimated mean value of graphene-flake lateral sizes of 253 ± 10 nm is sufficiently close to the DTC emulsion hydrodynamic diameter measurements of 266 ± 6 nm. This means that by assessing the dimensions of the exfoliated graphene flakes in the stabilized emulsion with relatively simple hardware, one can be fairly confident in what will be found in the composition of the coating applied to the fibres.

**Figure 9:**
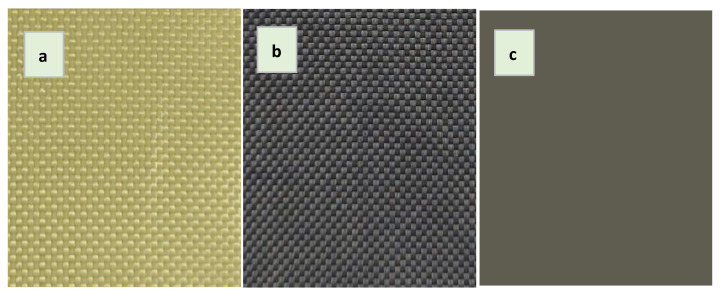
Colour of pristine fabric (**a**), graphene functionalized fabric (**b**) and fabric quantified in CIELAB colour space L*a*b* (**c**).

The colours of pristine and modified samples visualized and quantified by using the CIELAB colour space L*a*b* mode (Figure 9) offer another opportunity to quickly evaluate the covering of the modifying coating and conduct a comparative analysis of the variants.

**Figure 10:**
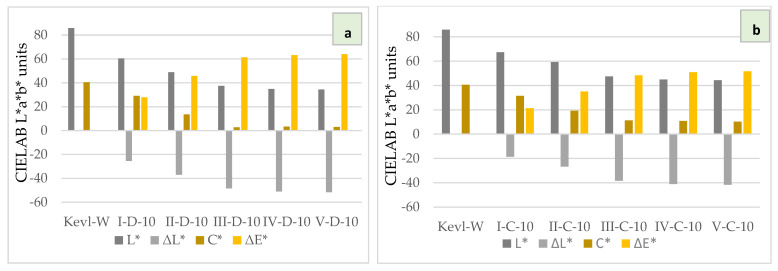
Quantified lightness difference (ΔL*), chroma (C*) and colour difference (ΔE*) of modified (I–V) and pristine fabric (Kevl-W) samples. (**a**) DMAc- and (**b**) Cyrene-based emulsions.

The graphs in Figure 10 show well how gradually increasing the number of deposited coatings of graphene flakes overlap Kevlar’s characteristic yellow in DMAc-based dispersion of sequentially deposited layers (Figure 10a) and in the case of Cyrene (Figure 10b). When comparing graphs in Figure 10a,b, it is not difficult to see that (1) in all DMAc-based coatings, graphene layer thickness increases about 25% faster (Figure 10a) than in Cyrene-based coatings (Figure 10b); (2) the fourth- and fifth-layer coatings increase both chroma (C*) and ΔL* and ΔE* values very little.

### 3.3. Comparative Analysis of the Ballistic Performance of Hybrid Soft BP Packages with Integrated Graphene-Modified Para-Aramid Fabric Layers

Based on the analysis of the morphology of graphene-modified surfaces and tracing the effect of the number of coating layers and their specificity on the structure of the coating, as well as taking into account the resource capacity in the BP package striking face (first group), we integrated three graphene-modified Kevlar fabric layers with triple coating.

During the ballistic test, 14 pieces of hybrid soft BP package variants with integrated layers of graphene-modified para-aramid fabrics in combinations with the different ballistic Kevlar textiles were shot. Data for the non-perforated BP packages is shown in Table 5.

In the first group (Table 5, rows 1–3), replacing 5 layers of UD XP with three layers of modified KM2-600 reduces the total number of layers, area density decreases by 8%, average BFS decreases by 10%, and its confidence interval decreases by two times. In the UD3 BP package, three graphene-modified layers are successively added to the 23 UD XP layers and optionally supplemented with two aerogel layers (Table 5, rows 1–3). BFS of UD3 does not exceed the allowed level, but has increased compared to both previous variants, and the area density has increased due to the aerogel layers. The relatively high BFS confidence interval suggests the unstable structure of the package, which arises from the fact that the layers of the aerogel were placed last. It is possible that closing a package with a single UD XP layer would stabilize the package architecture.

In the second group (rows 4–6) variant PE, PE-IT and PEUS UHMWPE layers vary from 32 to 38, integrating three modified KM2+ 440 layers (row 5) or three modified KM2 600 layers into the arrangement. In the third variant of this group, 32 layers are sorted behind three modified KM2 600 layers. The back layer of the package is formed by the trauma board by increasing the areal density of the package. Compared to the two previous variants of this group, both BFS and its confidence intervals (row 6) are significantly reduced.

In the third group (rows 7–9), the base layers are formed from KM2+ 440 fabric, with 38 layers in the reference variant IT-38 (row 7) and 35 layers in the IT-K variant (row 9). In both the reference and IT-IT versions, the BFS exceeds the value of BFS permissible for the relevant level of protection, with a decrease in IT-IT package areal density by 25% and penetration depth by 10% The structure of the IT-38 and IT-IT packages is deformed as a result of impact; the fabric massively breaks down to fibres over a large area. Stabilizing the layers of the package structures by stitching through, creating 12- and 24-layer blocks, respectively, and replacing the modified KM2+ 440 layers with the modified KM2 600 layers, produces a facilitated flexible package with a more stable structure (row 10). 

In the fourth group (rows 10–11), the base layers are formed from Kevlar^®^ XP™ K520 whose architecture consists of 2 layers of fibres in a +45°/−45° orientation, which provides the necessary stopping power, eliminating standard ballistic threats to the body with a smaller number of layers. Ballistic protection package 3DK (row 11) supplemented with two layers of aerogel. In package 3DKM2 (row 10), instead of one Kevlar^®^ XP™ K520 layer, an optional three graphene-modified KM2 600 layers are incorporated. As a result, a stable structure of the hybrid BP package is formed, which provides a decrease in penetration depth by 44%; the areal density of the package does not change significantly.

Analysing the test results, it can be concluded that the BFS depths of only two samples (No 7 and 8) show penetration depth (BFS) values above those provided by the standard, but nine samples fit the standard requirements. The best results are obtained for samples No 10 (3DKM2: Du Pont™ Kevlar^®^ XP™ K520, Modified KM2-600, Airloy^®^ HR)—18.1 mm, No 6 (PEUS: UHMWPE: SFPE03, Modified KM2-600, Trauma board)—23.1 mm, No 11 (3DK: Du Pont™ Kevlar^®^ XP™ K520, Airloy^®^ HR)—26.3 mm. The other six samples showed results of 26.3–39.4 mm.

### 3.4. Shot Records by Pressure Sensors

To analyse the ability of the graphene-modified para-aramid fabrics layers of the panel to stop the bullet, changes in the resistance of the underlaying piezoresistive pressure sensor at the moment of impact of the bullet were recorded. Table 6 describes the sample designations and bullet parameters.

**Figure 11:**
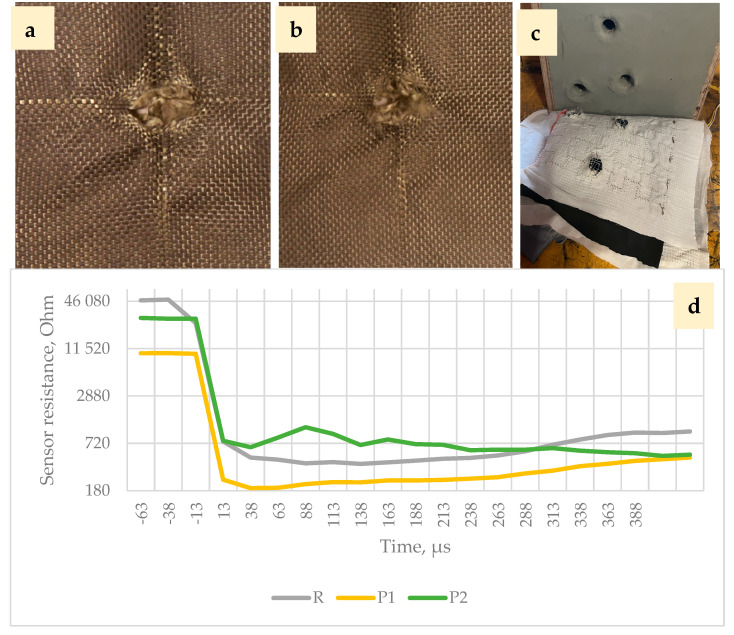
First P2 perforated functionalized KM2 600 fabric layer striking face (**a**) and exit face (**b**), and matrix pressure sensor with BFS (**c**). Sensor resistance changes that represent the perceived bullet impact energy behind the ballistic package are shown in (**d**).

**Figure 12:**
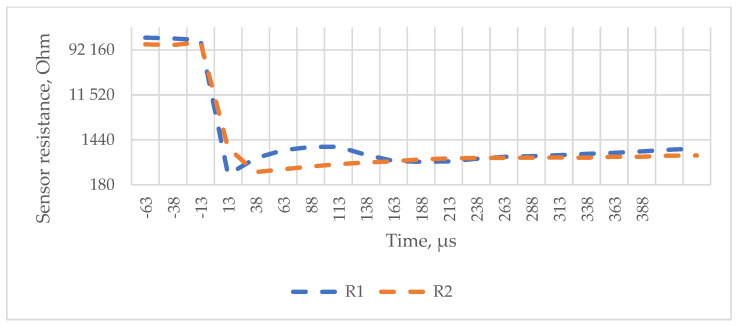
Data corresponding to two shots that perforated the reference BP packages.

**Figure 13:**
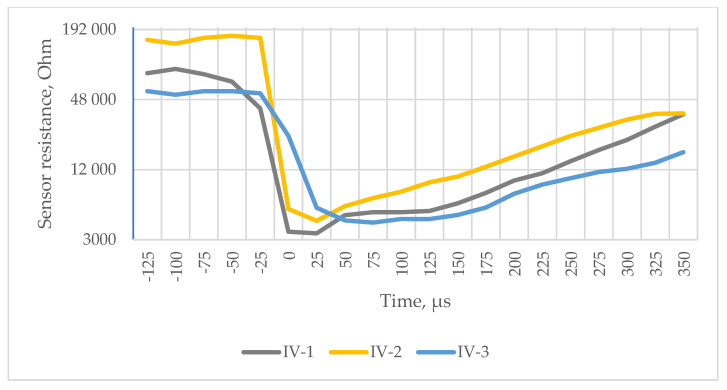
Knitted sensor test results of UD-reference samples (IV-1, IV-2, IV-3: shot numbers).

Figure 11d, Figure 12 and Figure 13 show signals measured by the pressure sensors during ballistic tests that represent the impact of the bullet.

The sensor activates and switches to data-recording mode when a certain voltage threshold value is reached, thus recording an event that corresponds to a single shot. After the data are downloaded, the sensor resets itself and is ready to record the next shot. While operating at a sampling rate of about 80 kHz, the sensor saves a “history” of 500 measurements that represent state preceding the threshold point and 4500 data points after the threshold, which in total correspond to a time window of about 6 ms. The threshold at which the sensor activates is around 3095 ohms in these tests. The zero point on the time axis corresponds to the point at which the threshold is reached.

As shown in Figure 11, Figure 12 and Figure 13, resistance decreases as the sensor piezoresistive layers are compressed (the sensor incorporates mesh spacers that spatially separate surfaces of the piezoresistive fabrics from each other to reduce resistance and power consumption at the resting state). Impact causes a decrease in contact resistance and compression of the piezoresistive layers, which further reduce the resistance.

In the P1 BP package, the impacted bullet bounced from the first fabric layer, and the energy of the impact received decreased very sharply (Figure 11d, yellow graph). Threads of the first and two following modified layers were not broken, but were pushed sideways, forming a hollow bullet imprint. The fourth UHMWPE layer cavity walls have thinned. 

Comparing the first impacted layer striking face and exit face in the P2 BP package (Figure 11a,b), compaction of the fabric structure in a diamond shape around the area of the projectile hit is seen. The light bands are formed by one to three primary yarns that dissipate the energy during the first μs, pulling out the threads from the fabric structure; the amount is influenced by the kinetic friction coefficients of yarn-to-yarn, fibre-to-fibre and projectile-to-yarn. The main mechanism of damage aside from the pulling out of primary threads from the fabric structure is a few broken threads, yarn separation into fibres and fibre fibrillation affected by the radial resistance of yarns and fibres to transverse deformations. Impact response of package P2 (Figure 11d, green line) shows improvement of the resistive force compared to P1 during the first 10 µs due to graphene coating and contributes along with the next two perforated modified layers to the greater impact energy absorption tendency of the whole package. 

The reduction in “baseline” resistance (Figure 11d) at rest state in subsequent shots is explained by the fact that before the first shot, while the sensor is not damaged, all of the layers are separated from each other by spacer layers, but right after the first shot, the sensor is damaged, the layers are compressed at the point of impact, additional contact points are created between the piezoresistive textile layers and between the conductive tracks, and a larger parasitic current begins to flow, which results in higher voltage readings; therefore, the resistance of the sensor decreases.

The analysis of the dynamics shown on the sensor signal curve after the impact makes it possible to analyse the characteristics of the impact on Kevlar fabric layers.

It can be concluded that if the panel is shot through, a smaller amount of energy is dissipated sideways, the impact is more concentrated, and the impact event itself is shorter (the resulting integral aggregate is smaller). If the bullet is stopped, then the entire “impact” is slower and the sensor registers the changes for a longer period of time (the resulting integral aggregate is larger).

A comparison of the graphs obtained in the first 300 μs with both types of sensors (Figure 11d, Figure 12 and Figure 13) shows that the sensor resistance recorded as a result of the impact decreases practically instantaneously to a minimum level, confirming the hypothesis put forward that textile layers with increased resistance should be inserted into the first layers of the ballistic package compared to the package layers for transferring the impact power to an increasingly wide area. The resulting graphs also allow us to compare the variants because they obviously correlate with obtained BFS measurements. In future studies, these tests should be applied to a wider extent by adapting the capability of sensors and methodology to obtain data with a sufficient resolution, especially in the first µs of impact.

## 4. Conclusions

A number of topical problems had to be solved in the stages of the technological sequence to develop processing methods and optimizing processing parameters in accordance with the processing specifics of aramid textiles and to achieve the desired properties of modified ballistic fabrics, including the provision of coating adhesion to the surface to be modified. 

In this research, as a result of theoretical and experimental studies, the main problem solved is the development of an original method of applying a graphene-containing coating to the fibres of para-aramid fabric by ensuring adhesion of the coating with the surface of para-aramid fibres. The developed method is also applicable to modify polyamide (nylon 6, nylon 66) fabric fibres with a graphene-containing nano-scale coating. The method includes several separate original solutions: (1) For graphene exfoliation from graphite flakes, liquid media constituents compatible with para-aramid and graphene properties were selected and their percentages experimentally evaluated; (2) laboratory technology was developed for obtaining a stable graphene-containing emulsion from graphite flakes; (3) a method was developed for applying graphene-containing coating to the surface of para-aramid fabric fibres; (4) Combining solutions 1–3, the technological sequence ‘graphite flakes–graphene-containing emulsion–para-aramid ballistic fabric modification–aramid fabric with functional fibre surface modification’ was created, excluding graphene extraction and re-dispersion processes with high resource consumption. There are no reports that such integrated studies have been carried out so far.

Based on the comparative analysis of existing know-how on the mechanism of bullet impact propagation in soft ballistics panel (BP) package layers in this research explored the possibility of improving soft BP performance by creating a hybrid package and incorporating three to four graphene-modified aramid (Kevlar^®^ KM 600 un Kevlar^®^ KM+ 440) layers into it in combination with both para-aramid-based ballistic materials (Kevlar^®^ KM+ 440, UD XP, Kevlar^®^ XP™ K520) and high-performance polyethylene (UHMWPE) sheets. Results of the comparative ballistic tests show that inclusion of graphene-modified textile layers in the hybrid BP package can reduce both the total number of layers in the package and/or the penetration depth (PD). 

A three-layer piezoresistive matrix-type pressure sensor made of textile materials was developed to remotely determine the point of bullet impact and the type of impactor (by measuring changes in the resistance of the sensor and inferring impactor’s speed and mass by analysing the registered signal). The pressure sensor provides information on changes in electrical resistance of the piezoresistive layers at the moment of bullet impact behind the ballistic package. Future studies will focus on developing aggregate indicators that will enable us to establish correlation with the performance of the protective layers.

The reduction in the areal density of the BP package is limited by the effects of the interaction of the type, mass and velocity of the bullet with the specific composition of the package and its areal density, and each combination corresponds to the areal density of the BP package within certain limits. If areal density of the BP package is too low under the action of a bullet impact, the structure of the BP package becomes destroyed in the first μs, so the total resistance ability of the BP package is no longer sufficient.

Evaluation of graphene-containing dispersion or emulsion ξ-potential, which is currently not very popular, is crucial to assess the stability of the established/planned composition. This can save recourses for development and evaluation.

To scale up technology from the lab, it is important to link methods and tools for process progress and quality control. It is important to try to find an opportunity to replace sets of very expensive equipment, the operation, maintenance and interpretation of the results of which require highly qualified personnel with various skill sets and knowledge, with simpler sets and methods that provide quickly obtainable, easy-to-read information.

## Figures and Tables

**Table 1:** Solvent selection performance criteria.

Substance	δD	δP	δH	Hansen Distance, MPa^0.5^	Surface Tension, mJ/m^2^	Dynamic Viscosity, 25 °C, cP
Graphite	18	9.3	7.7		46.7	
DMAc	16.8	11.5	9.4	3.7	32.4	0.9
Cyrene	18.8	10.6	6.9	2.2	33.6	14.5
TEA	17.3	7.6	21		45.9	24.1 *,16.2 **

* 20 °C, ** 30 °C.

**Table 2:** Trisodium citrate chemical structure and properties.

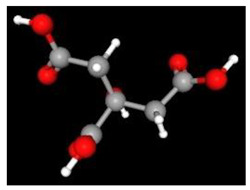 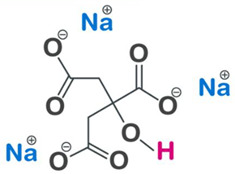	**Properties of Sodium Citrate (NaCi)**
	Granular crystals/crystalline powder
	Melting point > 300 °C
	Chemically stable
	Low reactive
	Hydrogen bond acceptor (**7**)
	Hydrogen bond donor (**1**)
	Stabiliser
	Emulsifying agent
	Inorganic and/or organic substances carrier
	Porous matrix
	pH regulator
	Non-toxic
Na^3^C^6^H^5^O^7^ [34]	Fully biodegradable

**Table 3:** Relative percentage of dispersion components.

	Graphite Flakes	DMAc	TEA	Sodium Citrate
	100 mg/mL			130 mg/mL
Wt.,%	7.3%	68.2%	15.1%	9.5%

**Table 4:** Comparative parameters ± SE characterizing the stability of the emulsions and hydrodynamic diameters of the graphene particles.

Hydrodynamic Diameter, nm		Polydispersity Index, %		Diffusion Coefficient, µm²/s		Zeta (ξ) Potential, mV		Electrophoretic Mobility, µm·cm/Vs		Conductivity, mS/cm
Mean	± SE	Mean	± SE	Mean	± SE	Mean	± SE	Mean	± SE	
**DTC**	Viscosity 1.89 mPa·s									
266.2	6.1	18.3	4.8	0.74	0.32	−37.9	1.15	0.58	0.02	0.013
**DTC-b**	Viscosity 2.17 mPa.s									
576.4	18.4	22.3	5.4	0.32	0.04	−35.1	0.02	-	-	0.010
**CTC**	Viscosity 231 mPa.s									
261.6	67.2	34.6	11.7	0	-	-	-	-	-	-
**CTC-b**	Viscosity 99.4 mPa.s									
353.7	132.7	57.4	54.7	0	-	-	-	-	-	-

**Table 5:** Comparative hybrid BP packages ballistic test results.

No	Designations	Materials	No of Layers	BP Areal Density, G/m^2^	BP Weight, G		Bullet Velocity, m/s		Starting Energy of Bullet, J		Penetration Depth, mm	
					Front	Back	Mean	±	Mean	±	Mean	±
1	UD-R	Aramid UD XP	29	5822	931	990	433	4	755	14	31	2
2	UD-E	Aramid UD XP	24	5394	863	917	433	8	754	27	29	1
		Modified KM2-600	3									
3	UD3	Aramid UD XP	23	5331	853	906	434	5	758	17	35	4
		Modified KM2-600	3									
		Airloy® HR	2	6153	984	1046						
4	PE	UHMWPE BP AM	38	5872	940	998	432	1	749	3	34	4
5	PE-IT	UHMWPE BP AM	33	5085	814	864	435	1	760	4	33	9
		Modified KM2+ 440	3									
6	PEUS	UHMWPE: SFPE03	32	5806	929	987	434	7	758	25	23	2
		Modified KM2-600	3									
		Trauma board	1	6430	1029	1093						
7	IT-38	KM2+ 440 310L	38	5094	815	866	429	7	740	24	53	16
8	IT-IT	KM2+ 440 310L	33	4801	768	816	429	8	739	29	48	6
		Modified KM2+ 440	5									
9	IT-K	KM2+ 440 310L	35	5085	814	864	436	2	763	7	36	3
		Modified KM2-600	3									
10	3DKM2	Kevlar® XP™ K520	9	5968	955	1015	434	6	758	21	18	1
		Modified KM2-600	3									
		Airloy® HR	2									
11	3DK	Kevlar® XP™ K520	10	6068	971	1032	434	5	758	16	26	5
		Airloy® HR	2									

**Table 6:** Designation of samples and bullet parameters.

Designations	Layer No	Bullet Weight, g	Bullet Speed, m/s	BFS, mm	Signature D, mm
R1	38 PE AM	8	420	Fired through	-
R2	38 PE AM	8	365	Fired through	-
R reference sample	38 PE AM	7	365	23.23	44
					61
P1, KM2-600	33 PE AM	8	356	25.45	44
	3 modif., KM2-600				55
P2, KM2-600	33 PE AM	8	377	27.67	56
	3 modif., KM2-600				47

## Data Availability

The original contributions presented in the study are included in the article, further inquiries can be directed to the corresponding author.
